# Association of chromosome 2q36.1–36.3 and autosomal dominant transmission in ankylosing spondylitis: results of genetic studies across generations of Han Chinese families

**DOI:** 10.1136/jmg.2009.066456

**Published:** 2009-05-04

**Authors:** J Gu, J Huang, C Li, L Zhao, F Huang, Z Liao, T Li, Q Wei, Z Lin, Y Pan, J Huang, X Wang, Q Lin, C Lu, Y Wu, S Cao, J Wu, H Xu, B Yu, Y Shen

**Affiliations:** 1 Third Affiliated Hospital of Sun Yat-sen University, Guangzhou, China; 2 The Institute of Genomic Medicine of Sun Yat-sen University, Guangzhou, China; 3 Chinese PLA General Hospital, Beijing, China; 4 The Institute of Basic Medical Sciences, Chinese Academy of Medical Sciences & Peking Union Medical College and Chinese National Human Genome Research Centre, Beijing, China

## Abstract

**Background:**

Ankylosing spondylitis (AS) is a chronic, potentially crippling, spondyloarthropathy with strong genetic components affecting approximately 0.3% of the population. Its exact genetic mechanism and mode of transmission, however, remains obscure.

**Methods and results:**

The authors conducted a genome wide scan on 75 individuals across multiple generations of three Han Chinese families affected with AS. Segregation analysis and pedigree investigation suggested an autosomal dominant inheritance. Pairwise logarithm of odds (LOD) scores were calculated using LINKAGE package for the obtained genotypes. High resolution mapping was then performed based on markers with significant LOD scores. To minimise the number of crossovers in each family, haplotype were constructed and assigned. Two of the pedigrees shared one candidate region for AS on 2q36.1–2q36.3 spanning 6-cM (maximum heterogeneity LOD score of 12.41 at marker D2S2228), while the other showed strong linkage to the HLA-B region.

**Conclusions:**

This is the first report which proposes one of the new genetic models of autosomal dominant transmission in AS. The breakthrough in the identification of linkage to chromosome 2q36.1–2q36.3 and the HLA-B region highlights the future potential of more comprehensive genetic studies of determinants of disease risk.

Ankylosing spondylitis (AS; MIM 106300) is a chronic, debilitating, inflammatory disorder that primarily affects the axial skeleton and frequently involves the peripheral joints, the attachments of ligaments and tendons to joints (the entheses), and also extra-articular structures. The overall prevalence of AS in the world population has been reported to be 0.2–0.9%.^1^ In the Chinese population, the pooled prevalence is not much different, and has been reported to be around 0.2–0.4%.^2^

The precise cause of AS is still unclear. The aetiology of the disease is predominantly determined by genetic factors, and environmental factors also play a role. Previous twin based studies estimate that disease heritability exceeds 90%.^3^ The recurrence risk ratio for siblings (λs) of AS has been reported to be as high as 82.^4^ A consistent strong linkage to chromosome 6, including the HLA-B region, has been recognised for decades. Among the Asians, 5–10% of the population is B27 positive. Yet, only 1–5% of these B27 positive individuals develop AS. B27 can explain no more than 30% of the overall genetic risks of AS.^5^ The mechanistic hypotheses of B27 causing AS are also not well established.^6^ Recently, two new loci related to AS, aminopeptidase regulator of TNFR1 shedding 1 (ARTS1) and the interleukin 23 receptor (IL23R), were independently reported in a North American cohort by 14 436 non-synonymous SNPs (nsSNPs) screen, confirming an AS related genetic disorder outside HLA-B27 through case–control association study.^7^

Genome-wide linkage scans have also implicated several additional loci outside the major histocompatibility complex (MHC) region.^8^ ^9^ ^10^ The decoding of the molecular aetiology of AS is contingent on the identification and characterisation of these non-MHC susceptible genes.

## Subjects and methods

### Subjects and ascertainment of the pedigrees

Peripheral blood samples were obtained from a total of 75 individuals (25 AS patients and 50 unaffected subjects) representing three to four generations of three large Han Chinese families affected with AS. Each patient was assessed by at least two qualified rheumatologists, and the diagnosis was made according to the 1984 modified New York criteria of AS.^11^ Subjects were defined as unaffected individuals if they had no evidence of AS related clinical manifestations and radiographic or magnetic resonance imaging changes. Informed consent was obtained from each subject enrolled into the study. The approval of the local ethical committee was also obtained before initiating the study.

### Segregation analysis

Before the model based genome wide linkage analysis of the three pedigrees with AS, segregation analysis was performed to investigate the presence of major type effects and segregation patterns within the pedigrees using the SAGE program SEGREG for binary phenotypes.^12^ Regressive multivariate logistic models for binary traits were used to investigate the segregation pattern within the pedigrees.^13^ In this model, the marginal probability (called susceptibility) that any pedigree member has a particular phenotype is the same for all members who have the same values of any covariates in the model and is given by the cumulative logistic function:


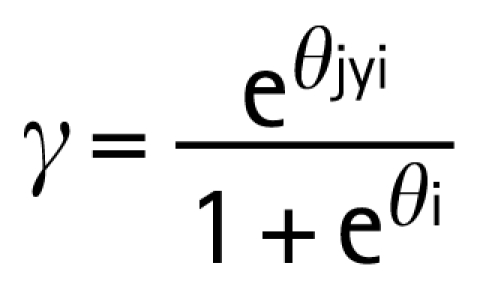


where yi is the trait value for the ith individual and is 1 for an affected individual and 0 for an unaffected individual; and θi is the logit of the susceptibility for the ith individual, which depends on the major type (u = AA or AB or BB) and covariates xi1, xi1,…, xip:


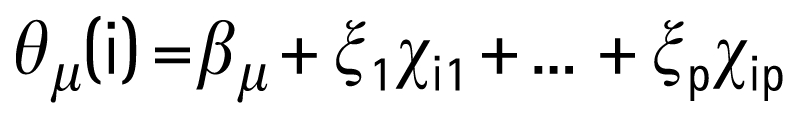


The nuclear familial residual association parameter (r), which is analogous to the correlation parameter in regressive models for continuous traits,^14^ is a second order correlation and is incorporated into the models to account for residual polygenic and common environmental effects. We assumed no spouse correlation and equal parent–offspring and sib–sib correlation throughout the analyses. Ascertainment bias was corrected by modelling the simplex sampling scheme from which the pedigree was recruited. Hypotheses were assessed by the likelihood ratio test, under the assumption that the negative of twice the difference in natural logarithms for hierarchical models follows a χ^2^ distribution.^15^

### DNA extraction and genotyping

Genomic DNA was extracted from venous blood according to established protocols.^16^ Genome-wide scans were performed on pedigree A and C. Genome-wide scans using 382 fluorescent microsatellite markers (ABI Prism Linkage Mapping Sets, Version 2.5) located on all autosomes were performed. Genotyping was performed on an Applied Biosystems (ABI) 3700 automated DNA sequencer. Each multiplex PCR reaction was performed according to standard instructions. Gene mapper 3.0 (Applied Biosystems, Foster City, California, USA) was used for data collection and microsatellite allele analysis. All genotypes were checked for Mendelian segregation in pedigrees.

### Linkage analysis

We conducted both parametric and non-parametric linkage analysis in all three pedigrees (A, B, and C). For parametric linkage analysis, pairwise two-point (logarithm of odds) LOD scores were calculated using the LINKAGE package. An autosomal dominant model was assumed and, in view of the prevalence of the disorder, the frequency of the abnormal allele was set at 0.003 with an assumed penetrance.^1^ ^2^ Allele frequencies for the microsatellite markers were set at 1/n (n =  number of alleles). The recombination frequency (θ) was stated as equal for both sexes. Heterogeneity LOD (hLOD) scores were computed by combining results per family with use of standard formulas HLOD = log10 (maxLR), where maxLR is maximised with respect to α, the proportion of the linked families, yielding maximum likelihood estimate.

### High resolution mapping

High resolution mapping was performed in all three pedigrees (A, B and C). Marshfield markers were selected according to marginal negative markers with priority given to marker heterozygosity based on the initial genome scan results. We constructed and assigned the shared haplotype in families for potential region.

## Results

**Figure 1:**
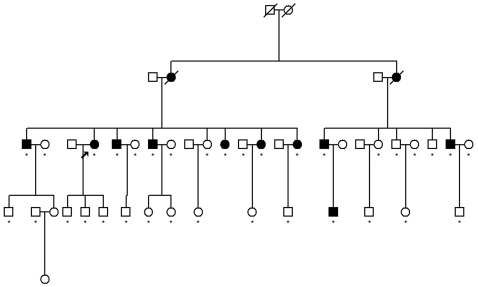
Family structure of pedigree C. Star mark represents individuals with collected DNA samples conducted in genome wide scan and fine mapping. No star mark represents individuals without collected DNA samples who were unwilling to donate their blood. The proband is marked with an arrow.

**Figure 2:**
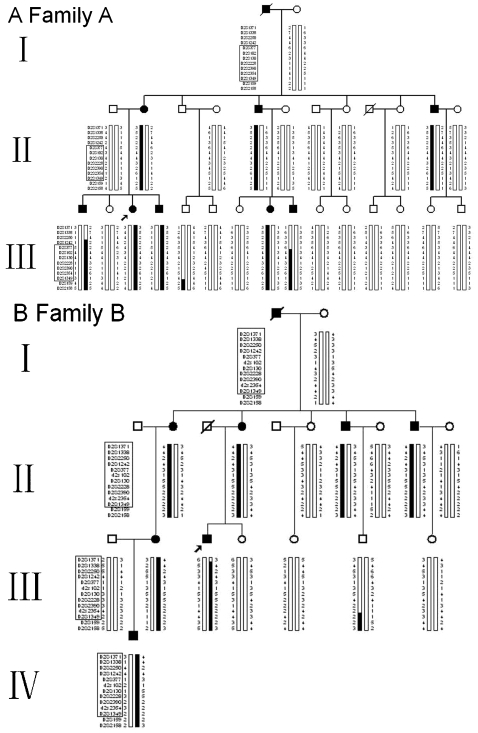
Shared haplotype of family A and B. Marker haplotypes on chromosome 2q36.1–2q36.3 that are linked to ankylosing spondylitis (AS) are indicated by black bars. Marker positions were obtained from Marshfield (http://research.marshfieldclinic.org/genetics/GeneticResearch/compMaps.asp). Microsatellite markers are listed at left from centromere to telomere (top to bottom). The proband was marked with an arrow. Haplotypes were interpreted by minimising recombinants. In each haplotype pair, paternal haplotype is to the left and maternal to the right.

The distribution of affected AS patients among the three pedigrees and their family structure are shown in figs 1 and 2. In the 25 AS patients of the 3 families, 15 are male and 10 are female (male: female ratio 3:2). The disease duration (mean (SD)) is 9.82 (5.80) years. The onset age (mean (SD)) is 22.45 (6.21) years, ranging from 9–31 years. In clinical manifestations, all patients have sacroiliac and spinal involvement. Among them, nine patients had peripheral joint involvement and 12 patients had enthesitis in three families, respectively. In family A, three patients had hip involvement. None of the affected individuals had a history of uveitis.

Complex segregation analysis was employed to investigate the inheritance pattern within the pedigrees. Parameter estimates for six different models of family C are shown in the table 1.

**Table 1:** Multivariate logistic maximum likelihood estimates of segregation models for inheritance of ankylosing spondylitis in family C

	Multifactorial only	Multifactorial+commingled	Multifactorial+Mendelian	Multifactorial+free τ’s	Multifactorial+dominant	Multifactorial+recessive
qA	(1.0)	5.13×10^−3^	0.68	0.84	0.70	0.97
τ_AA_	…	…	(1.0)	0.0	(1.0)	(1.0)
τ_AB_	…	…	(0.5)	1.0	(0.5)	(0.5)
τ_BB_	…	…	(0.0)	0.0	(0.0)	(0.0)
Susc_AA_	…	0	7.67×10^−30^	7.36×10^−35^	4.41×10^−22^	8.49×10^−55^
Susc_AB_	…	0.14	7.61×10^−26^	0	4.41×10^−22^	0.50
Susc_BB_	0.17	0.14	0.51	0.54	0.56	0.50
ρ_resid_	1.67	8.08	4.00	4.02	4.06	3.87
-2lnL	47.16	32.15	26.38	22.36	26.44	28.13
Akaike’s AIC	51.16	42.15	36.38	32.36	34.44	36.12

Test H_01_: no major gene effect, χ^2^ = 20.78, df = 3, p = 1.17×10^−4^.

Test H_02_: no transmission of major gene effect, χ^2^ = 9.79, df = 3, p = 0.02.

Test H_03_: Mendelian transmission, χ^2^ = 4.02, df = 3, p = 0.26.

Test H_04_: Mendelian transmission and dominant inheritance, χ^2^ = 0.06, df = 1, p = 0.81.

Test H_05_: Mendelian transmission and recessive inheritance, χ^2^ = 1.75, df = 1, p = 0.19.

The null hypothesis of no major gene effect was assessed by comparing the model containing multifactorial inheritance alone (H01: qA = 1) with that containing both a Mendelian major gene and multifactorial inheritance (HA1: 0<qA<1; τAA = 1.0, τAB = 0.5, τBB = 0.0). The model with both effects has three additional parameters (qA and two susceptibilities), so the likelihood ratio statistic (χ^2^) has three degrees of freedom (df). The null hypothesis of no transmission of major gene effect was tested by comparing the commingled model (H02: qA = τAA = τAB = τBB) with the model in which all transmission probabilities were estimated (HA2: qA≠τAA≠τAB≠τBB, 3 df). The null hypothesis of Mendelian transmission was tested by comparing the mixed Mendelian model (H03: τAA = 1.0, τAB = 0.5, τBB = 0.0) with a model where all transmission probabilities were estimated (HA3: free τ’s, 3 df). The null hypothesis of dominant Mendelian inheritance (H04) was tested by comparing the model of the mixed Mendelian model (the general model) with the mixed model (SuscAA = SuscAB≠SuscBB, 1 df). The null hypothesis (H05: SuscAA≠SuscAB = SuscBB) of recessive Mendelian inheritance was tested in similar way. In family C, the hypothesis of no major effect was rejected (χ^2^ = 20.78, df = 3, p = 1.17×10^−4^), and the hypothesis of no transmission of the major effect was also rejected (χ^2^ = 9.79, df = 3, p = 0.02). As expected, the hypothesis of Mendelian transmission was not rejected (χ^2^ = 4.02, df = 3, p = 0.26). Both dominant and recessive Mendelian inheritance models were not rejected with p = 0.81 and 0.19, respectively.

These results clearly support the conjecture that a putative major gene was segregating in the large and extended pedigree in the manner of autosomal dominant Mendelian inheritance. In family A and B, the hypothesis of no major effect was not rejected (χ^2^ = 3.97, df = 3, p = 0.27), but both the hypothesis of dominant and recessive Mendelian inheritance models were not rejected with p = 0.44 and 0.35, respectively. This discrepancy between the three tests might due to the fact that the two models for H01 are not hierarchical with each other and thus the test for H01 is less powerful, although such a test was extensively used in the segregation analysis. The results of the later two tests clearly support the conjecture that a putative major gene was segregating in these two pedigrees, and maybe it is more likely to be segregating in the manner of autosomal dominant Mendelian inheritance. All the LOD scores were evaluated at recombinant fraction from 0.0 to 0.4 with the penetrance from 0.56 to complete penetrance (LOD scores calculated with the penetrance of 0.56 based on our segregation analysis result of family C are shown in table 2).

**Table 2:** Two-point logarithm of odds (LOD) scores for chromosome 2 markers in two Han Chinese pedigrees with ankylosing spondylitis

Distance (cM)	Marker	Het	Family A LOD score					Family B LOD score					hLOD (α, θ)	p Value
			θ					θ						
			0	0.1	0.2	0.3	0.4	0	0.1	0.2	0.3	0.4		
215.25	D2S1371	0.78	−2.57	0.84	0.91	0.68	0.32	−1.14	0.57	0.51	0.33	0.11	3.27 (1.0, 0.2)	0.011
215.78	D2S1338	0.87	−2.92	0.84	0.91	0.68	0.32	0.53	0.44	0.33	0.21	0.09	2.95 (1.0, 0.1)	0.015
216.31	D2S2250	0.78	−2.78	0.84	0.91	0.68	0.32	2.14	1.71	1.25	0.78	0.31	5.87 (1.0, 0.1)	6.11E-04
218.45	D2S1242	0.88	0.39	1.75	1.47	1.02	0.48	2.38	1.96	1.49	0.97	0.44	8.54 (1.0, 0.1)	3.57E-05
220.59	D2S377	0.71	0.44	1.79	1.52	1.06	0.5	1.15	0.9	0.66	0.42	0.2	6.19 (1.0, 0.1)	4.32E-04
222.2	D2S102	0.86	2.28	1.88	1.44	0.95	0.42	1.03	0.81	0.59	0.38	0.18	7.62 (1.0, 0.0)	9.45E-05
222.73	D2S130	0.75	0.98	0.83	0.66	0.47	0.25	1.41	1.14	0.85	0.52	0.2	5.50 (1.0, 0.0)	9.08E-04
224.33	D2S2228	0.78	3.26	2.7	2.07	1.39	0.66	2.13	1.71	1.26	0.78	0.32	12.41 (1.0, 0.0)	6.29E-07
225.67	D2S2390	0.6	0.83	0.66	0.49	0.32	0.15	0.12	0.1	0.07	0.03	0.01	2.19 (1.0, 0.0)	3.70E-02
227.54	D2S2354	0.8	3.26	2.7	2.07	1.39	0.66	2.08	1.73	1.33	0.87	0.39	12.30 (1.0, 0.0)	7.08E-07
228.01	D2S1349	0.57	1.24	1.0	0.73	0.44	0.15	1.04	0.85	0.64	0.43	0.21	5.25 (1.0, 0.0)	1.19E-03
228.61	D2S159	0.77	0.85	0.67	0.49	0.31	0.14	0.63	0.49	0.35	0.2	0.09	3.41 (1.0, 0.0)	9.03E-03
229.14	D2S2158	0.77	2.59	2.23	1.75	1.2	0.59	2.08	1.73	1.33	0.87	0.39	10.75 (1.0, 0.0)	3.53E-06

All the LOD scores were evaluated at recombinant fraction from 0.0 to 0.4 with the penetrance of 0.56. The maximum heterogeneity LOD score was obtained at marker D2S2228.

Parametric linkage analysis was performed for the three pedigrees for the autosomal recessive model with the assumed penetrance derived from segregation analysis to complete penetrance, and the recessive inheritance model was excluded based on the unfavourable two-point LOD scores in the tests.

**Figure 3:**
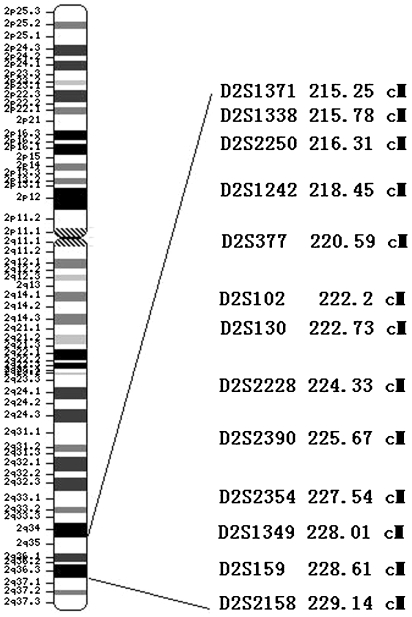
Scale map of the 13 fine mapping markers of pedigree A and B. Scale map of markers on chromosome 2 for fine mapping of the ankylosing spondylitis loci. Thirteen markers were selected from 2q35–36.3 and one marker was used in the genome-wide scan. The distances between markers were plotted according to sex averaged distances determined with CEPH pedigree data.

Initial analysis data using LINKAGE revealed a positive linkage LOD score of 2.28 at marker D2S126 (θ = 0) in family A, while a negative LOD score of −4.72 at this marker was obtained in pedigree C. Additional markers within 10 cM around marker D2S126 (221.13 cM) from the Marshfield website were selected to extend fine mapping in pedigree A and B (fig 3). The heterozygosity of these markers and LOD scores and heterogeneity LOD scores of the families A and B, targeted in the shared region, are available in table 2. By analysing individuals in which recombination had occurred in both families, we narrowed the region to a 6 Mb region on 2q36.1–2q36.3 between markers D2S377 and D2S1349 (fig 2). The maximum heterogeneity LOD score in pedigree A and B reached 12.41 (family A maximum LOD score is 3.26 and family B maximum LOD score is 2.13) at marker D2S2228.

Based on linkage to chromosome 6 at marker D6S422 (LOD 1.76, θ = 0) in initial genome scan in pedigree C, we selected five Marshfield markers located between 42.27 cM and 50.75 cM around the HLA-B region, including D6S2439, D6S273, D6S1666, D6S1583 and D6S1051, to extend fine mapping in pedigrees A, B and C. In pedigree C, the highest LOD score 4.02 was obtained at D6S273 (θ = 0). In pedigree A, the highest LOD score 2.67 was obtained at D6S1583 (θ = 0). In pedigree B, no linkage was observed for these fine mapping markers.

## Discussion

The inheritance mode of AS tested by sibling recurrent risk studies has been considered as oligogenic and multiplicative interaction among loci.^4^ Epidemiologic studies suggested a non-sex linked dominant heredity involvement,^17^ but until now no affirmative mode of inheritance has been proposed. Although AS has been widely considered to be a multifactorial genetic disease with a well accepted knowledge of linkage to the MHC loci, its role in the pathogenesis of AS has not been well established and existing studies revealed evidence of non-MHC loci. Meanwhile, subjects in previous genome wide scans conducted in western countries were mainly affected sib pairs (ASM) or nuclear families, without multi-generational large pedigrees recruited. In the present study, we have described for the first time that a new form of AS inheritance mode is autosomal dominant, based on not only pedigree investigation and segregation analysis, but also parametric linkage analysis with genome scans and fine mapping in these families. Among them, family A and B shared the new region located in 2q36.1–2q36.3, and the HLA-B locus was verified based on our original single large pedigree family C with the maximum LOD score of 4.02 at marker D6S273. We initially proposed an affirmative autosomal dominant inheritance in our three Han Chinese pedigrees as one of the genetic modes in this complex and genetically heterogeneous disease.

Brown *et al* conducted a genome scan in 1998 and revealed nominal linkage with a LOD score of 0.8 at marker D2S126,^5^ located in the region between D2S377 and D2S1349. Significant LOD score was not achieved due to either insufficient genetic information or heterogeneity between families. The studies using individual case or sib-pair samples are more vulnerable to the effects of heterogeneity. Therefore, the identification of disease loci is better accomplished with family based linkage studies. Herein, we approached this AS genome-wide scan research in the Chinese population using a collection of pedigrees with multiple affected individuals.

Previous genome-wide scans have suggested that the susceptible locus of AS is in the HLA region. Strong associations with HLA-B region have been identified by non-parametric linkage analysis and case–control association studies. However, the role of HLA-B as the principal genetic determinant of AS has been questioned and a handful of non-MHC loci have recently been evaluated and proven to be associated with AS.^18^ In our study, we observe linkage to chromosome 6 which contains the HLA-B locus in the genome-wide scan analysis and fine mapping of family C with two-point LOD score >3. This result initially confirmed linkage to the HLA-B region accompanied by a major gene transmission with autosomal dominant inheritance in the large pedigree of our study.

In conclusion, by parametric linkage analysis, we have confirmed linkage to the HLA-B region in one AS pedigree and reported a new locus in two AS pedigrees located on 2q36 for the first time with autosomal dominant transmission.
